# Sodium Alginate and Chitosan as Components Modifying the Properties of Inulin Hydrogels

**DOI:** 10.3390/gels8010063

**Published:** 2022-01-17

**Authors:** Anna Florowska, Adonis Hilal, Tomasz Florowski, Paulina Mrozek, Małgorzata Wroniak

**Affiliations:** Department of Food Technology and Assessment, Institute of Food Science, Warsaw University of Life Sciences, 02-787 Warsaw, Poland; tomasz_florowski@sggw.edu.pl (T.F.); mrozek-paulina@wp.pl (P.M.); malgorzata_wroniak@sggw.edu.pl (M.W.)

**Keywords:** polysaccharides, inulin, sodium alginate, chitosan, food ingredients, food structure, hydrogels

## Abstract

The aim of the study was to investigate the influence of addition of sodium alginate (SA) and chitosan (CH) on the properties of inulin hydrogels. Inulin hydrogels (20 g/100 g) containing various additions (0.0, 0.1, 0.3, and 0.5 g/100 g) of SA and CH were produced. The hydrogels’ properties were assessed based on the volumetric gel index, microstructure, yield stress, texture, stability, and color parameters. According to the findings, the inclusion of these polysaccharides had no influence on the gelation ability of the inulin solution. The physical properties of the hydrogels containing SA or CH differed from hydrogels containing only inulin (INU). The obtained microstructural pictures revealed that the addition of SA and CH resulted in the formation of hydrogels with a more compact, smooth, and cohesive structure. Consequently, they had higher yield stress, strength, and spreadability values than INU hydrogels. The addition of chitosan in comparison with sodium alginate also had a greater effect in strengthening the structure of hydrogels, especially at the level of 0.5 g/100 g. For example, the addition of this amount of SA increased the yield stress on average from 195.0 Pa (INU) to 493.6 Pa, while the addition of CH increased it to 745.3 Pa. In the case of the strength parameter, the addition of SA increased the force from 0.24 N (INU) to 0.42 N and the addition of CH increased it to 1.29 N. In the case of spreadability this increase was from 2.89 N * s (INU) to 3.44 N * s (SA) and to 6.16 N * s (CH). Chitosan also caused an increase in the stability of inulin hydrogels, whereas such an effect was not observed with the addition of sodium alginate. The gels with the addition of SA and CH also had significantly different values of color parameters. Inulin–alginate hydrogels were characterized by higher values of the color parameter a *, lower values of the color parameter b *, and in most concentrations higher values of the color parameter L * compared to inulin–chitosan hydrogels. Based on the collected data, it can therefore be concluded that through the addition of sodium alginate and chitosan, there is a possibility to modify the properties of inulin hydrogels and, consequently, to better adapt them to the characteristics of the pro-health food products in which they will be used.

## 1. Introduction

Recent trends in health-promoting food products have resulted in an increase in the number of studies investigating the use of healthier functional ingredients while keeping the quality attributes, such as texture, flavor, color, and nutritional values acceptable for the consumer [1]. Healthier food alternatives, including plant-based, fat-free, or sugar-free products, are often obtained by using biopolymers, such as proteins and saccharides, as structural components [2,3]. These biopolymers can be used to form aqueous three-dimensional structural networks known as hydrogels. Hydrogels have attracted over the years a significant interest in the biomedical and pharmaceutical sectors and are gaining more interest in the food sector due to the variety of possible applications, depending on their source, induction technique, and induction parameters [4]. Biopolymeric hydrogels have a great potential to excel as food quality enhancers, especially in terms of organoleptic perception and nutrient modification (calories management), as well as in bioactive substances’ targeted delivery and in food packaging [5]. Hence, when looking for healthy and nutritious food solutions, biopolymeric mixed hydrogels that combine both functional and health-promoting properties should be considered. Inulin hydrogels are an interesting example used in food products.

Inulin (INU) is a prebiotic, fructan-type polysaccharide with several functions, including fat mimicking, texture modification, and acting as a low-calorie sweetener [6]. Inulin is manufactured on a large scale from Asteraceae plants, such as chicory intybus (*Cichorium intybus*), Jerusalem artichoke (*Helianthus tuberosus* L.), and Dahlia (*Dahlia Cav*.) [7]. Regarding its chemical structure, it is composed of fructose molecules linked by varying lengths of β-(2/1)-D-fructosyl-fructose bonds, which are commonly terminated by a single glucose molecule connected by an ∝-D-glucopyranosyl bond [8]. It is vital to note that the degree of polymerization (DP) and the presence of branching both have a significant impact on the functional properties of inulin. Inulin can form crystallites that aggregate and trap a large quantity of water under certain parameters, such as temperature, pH, pressure, and shear forces [9]. High DP (greater than 23) inulin, also known as high-performance inulin, produces a gel structure by aggregation, altering the texture of the product and producing a fat-like mouth feel, while lower DP and branched molecules of inulin increase flavor and sweetness and are used to partially replace sucrose in food products [10]. A characteristic feature of inulin gels is that they are relatively weak. In some food systems this may be an advantage (e.g., fat imitation) but sometimes it would be more advantageous to strengthen the structure. Presumably, such an effect could be achieved by adding proteins or other polysaccharides [11]. Some of the potentially interesting polysaccharides that could be used for this purpose, due to their characteristics and health benefits, are sodium alginate and chitosan. Application of these polysaccharides to modify the properties of starch gels has already been presented in the literature [12]. In the available literature, there are also researches in which inulin, in concentrations in the range of 0.1–4 g/100 g, is a functional additive to chitosan or alginate gels, and the purpose of its use is to improve the properties of these gels or bio-films or microcapsule walls, which are formed from aqueous solutions of these polysaccharides [13,14,15,16,17]. However, the question of what effect the addition of SA and CH has on the characteristics of inulin gels is not known.

Alginates are natural, linear polysaccharides produced mainly from brown sea algae (*Phaeophyceae*), but they can also be synthesized extracellularly by bacteria such as *Pseudomonas aeruginosa*, *Azotobacter vinelandii*, or *Pseudomonas fluorescens* [18]. In their natural state they are found as a combination of salt compounds such as sodium, magnesium, and calcium alginate. Alginates are comprised of β-D-mannuronic acid (M) and ∝-L-guluronic acid (G) that are connected by a β-1,4-glycosidic bond and terminated by a carboxylic group residue. The M/G ratio and arrangement differ according to the species and the tissue from which they were extracted [19]. The capability of an alginate polymer to produce a gel structure is determined by the amount of mannuronic (M) and guluronic (G) acids present in the chain. Based on the spatial configuration and binding mechanism, the M and G blocks of alginate differ significantly [20]. Consequently, the addition of calcium to alginate, which includes numerous G-block, results in the formation of a gel due to the voids that occur between two G-blocks that are precisely equivalent to the size of the calcium ions. On the other hand, alginate with a higher number of M-blocks produces a soft, liquid-like gel structure [21]. Unfortunately, these structure are not stable and show low mechanical and barrier properties, which is why investigations are being carried out to combine sodium alginate with other biopolymers [22]. Sodium alginate (SA) also has numerous beneficial features, and it is widely utilized in the pharmaceutical, biomedical, and food sectors [21,23]. This is along with the functional properties of SA (thickening, stabilizing, and gelling agent) that can be utilized to enhance the structural properties of the food product. Alginate application offers some health benefits due to its anti-microbial, immunomodulatory, antioxidant, and prebiotic activity [24].

Chitosan (CH) is a polymer derived from the partial or full deacetylation of chitin, which is present in the exoskeletons of crustaceans (e.g., crabs and shrimps) and insects, as well as yeast and fungus cell walls [25]. In terms of chemical composition, chitosan is a linear polysaccharide consisting of variable quantities of 2-acetamido-2-deoxy-β-D-glucopyranose (N-acetylglucosamine) and 2-amino-2-deoxy-β-D-glucopyranose (D-glucosamine) structural units connected by β-1,4-glycoside bonds [26]. The gelation process of chitosan can occur by self-association or through interactions, such as hydrogen bonds, van der Waals forces, and ionic or hydrophobic interactions [27]. Unfortunately, using the native chitosan as a gelling agent is difficult because of its insolubility, formation of gel structure without aggregations, and poor mechanical properties. Therefore, chemical modification of chitosan and the addition of other polymers have been used to improve gels’ properties [15,16]. Because of its biocompatible, biodegradable, non-toxic, and non-allergenic properties, chitosan and its derivatives are utilized in a variety of sectors, including medicine, pharmacy, food, cosmetics, agriculture, environmental protection, and waste treatment [28]. Chitosan, in addition to its antimicrobial and antioxidation capabilities, has excellent emulsifying, thickening, and gelling properties, which are used to stabilize food structures. Chitosan can also provide dietary fiber as it is not hydrolyzed or digested in the gastrointestinal tract [29].

As the available literature does not include any broader studies on the possibility of modifying inulin hydrogels by adding the abovementioned polysaccharides, the aim of the research was to investigate the effects of sodium alginate and chitosan on the gelation ability and properties of obtained inulin hydrogels.

## 2. Materials and Methods

### 2.1. Materials

Inulin (INU) Orafti^®^ HPX was purchased from BENEO GmbH (Mannheim, Germany). The sodium alginate (SA) was purchased from Agnex (Białystok, Poland), and high molecular weight chitosan (CH) (CAS number 9012-76-4; MDL Number: MFCD00161512, Assay Deacetylation > 75%) was purchased from Sigma–Aldrich (Munich, Germany).

### 2.2. Induction of Inulin Hydrogels

The thermal induction technique was used to produce the hydrogels. The preparation of an inulin gel (INU, a control sample containing only 20 g/100 g inulin) involved dissolving inulin in water using a heated magnetic stirrer (80 °C, 1500 rpm for 10 min). In order to obtain alginate–inulin and chitosan–inulin gels, solutions (0.1, 0.3, and 0.5 g/100 g) of these polysaccharides (SA, CH) were prepared by dissolving the proper amount of additive in hot water (90 °C) using a heated magnetic stirrer (1500 rpm for 15 min). After dissolving the solutions were cooled down (80 °C) and inulin was added (20 g/100 g) and dissolved by continual stirring with a magnetic stirrer until a homogenous mixture was produced. The obtained solutions were poured into 50 mL beakers so that the weight of the sample was 50 g. The solutions were maintained for 24 h (at 8 °C) to develop the gels’ structure. Following this, the inulin hydrogels were evaluated in terms of volumetric gel index, microstructure, yield stress, texture, stability, and color parameters. The given values are the averages of three independent replicates.

### 2.3. Methods

#### 2.3.1. Volumetric Gel Index (VGI)

VGI is a parameter that expresses a gel structure’s capacity to develop. VGI is equivalent to 0% when the gel structure is not formed, and it is equal to 100% when the sample is entirely gelled. The volumes of the gels were measured while the gel remained in the beaker. The volumetric gel index (VGI) was calculated as follows [30]:

(1) $$\mathrm{VGI}=\frac{\mathrm{VG}}{\mathrm{VT}}\times 100\;\left[\%\right]$$

where VG—volume of formulated gel, and VT—total volume of sample.

#### 2.3.2. Microstructure

An electron scanning microscope (Hitachi TM3000, Hitachi, Japan) equipped with an energy dispersive spectrometer (EDS) and digital image recording was used to visualize freeze-dried hydrogels. Samples were applied to a carbon band and coated with a thin layer of gold at pressures of 100–133 Pa. The microstructure was analyzed under an accelerating voltage of 25 or 30 kV, and a magnification of ×3000. The hydrogel’s structure was graphically elaborated using the MultiScan v.18.03 program (Computer Scanning System, Warsaw, Poland).

#### 2.3.3. Yield Stress

A rheometer (DV3T, Brookfield, Middleboro, MA, USA) equipped vane spindle V74 with a torque range HA was used to measure the hydrogels’ yield stress (Pa). The rotation speed was adjusted to rise by 0.10 RMP continuously until the gel structure reached its flow limit. The stress measured at this angular velocity was interpreted as the yield stress of the investigated gel structure. The measurement data were examined using the software that was supplied with the rheometer.

#### 2.3.4. Textural Properties

A texture analyzer (TA.XT Plus, Stable Micro Mixtures, Surrey, UK) with a 5 kg load cell was used to perform the texture analysis (at 20 °C). The texture analyzer was equipped with a 0.5 cm diameter cylindrical flat probe (P/0.5R) to measure the hydrogels’ strength (N). The measurement speed was 1.0 mm/s, and the sample penetration depth was 8 mm (one third of the sample height). The gel strength was measured while the gel remained in the beaker. To measure the hydrogels’ spreadability (N * s), the texture analyzer was configured with the TTC Spreadability Rig. The measurement speed was 3.0 mm/s. The data were processed with the Exponent version 6.1.4.0 (Stable Micro Mixtures, Surrey, UK) equipment software.

#### 2.3.5. Physical Stability

The physical stability of the hydrogels was evaluated using the dispersion analyzer— LUMiSizer 6120-75 (L.U.M. GmbH, Berlin, Germany), which measures the intensity of transmitted near-infrared light in samples. The following parameters were utilized for this analysis: hydrogel volume 1.8 mL; wavelength 870 nm; light factor 1; 1500 rpm; experiment period 15 h 10 min; interval time 210 s; temperature 20 °C. The sample length was used to demonstrate stability as a space and time-related transmission profile. The data were examined using the provided software (SepView 6.0; LUM, Berlin, Germany), and the instability index was computed.

The background of this technique is based on subjecting of the samples to a centrifugal force while near-infrared (NIR) light illuminates the entire sample cell. A sensor connected to the SepView measures the intensity of transmitted light as a function of time and position over the entire sample length simultaneously [31]. Among other tests, this technique is used to analyze gels’ stability [32].

#### 2.3.6. Color Parameters

The color components in the CIEL * a * b * system were determined using the Minolta CR-200 colorimeter (Minolta, Japan; light source D65, and a measuring head hole of 8 mm). In the CIEL * a * b * system the L * color parameter (from 0 to 100) indicates the brightness of the studied sample—the higher the values, the brighter the sample [33]. The a * color parameter values reflect the proportion of red or green in the examined samples. Negative results indicate a green color, while positive results indicate a red color [33]. The b * color parameter values reflect the participation of blue or yellow color in the examined samples. Negative results indicate blue, and positive results indicate yellow [33]. The measurements were taken at the surface of the hydrogel samples. The parameter of total color difference ∆E was computed to determine the color differences between the control sample and the hydrogels with the addition of SA and CH. The total color difference was calculated as follows [34]:

$$\Delta E=\sqrt{{\left(L_{c}^{*}-L_{p}^{*}\right)}^{2}+{\left(a_{c}^{*}-a_{p}^{*}\right)}^{2}+{\left(b_{c}^{*}-b_{p}^{*}\right)}^{2}}$$

where $L_{c}^{*}$, $a_{c}^{*},\;$ and $b_{c}^{*}$ represent the color parameters of control INU hydrogels without SA or CH addition and $L_{p}^{*}$, $a_{p}^{*}$, and $b_{p}^{*}$ represents the color parameters of INU hydrogels with SA or CH addition.

The color difference between the samples can be estimated basing on the ΔE values [34]:

− If 0 < ΔE < 1—the color difference is determined as not noticeable to the observer.
− If 1 < ΔE < 2—only experienced observers can notice the difference in colors.
− If 2 < ΔE < 3.5—unexperienced observers also notice the difference in colors.
− If 3.5 < ΔE < 5—clear color difference in colors is noticed, and the observer notices two different colors (5 < ΔE).

#### 2.3.7. Statistical Analysis

The gathered data from three independent, experimental repetitions were statistically evaluated using the Statistica 13.3 (TIBCO Software Inc., Palo Alto, CA, USA) software. A one-way analysis of variance was performed to assess the significance of differences in the average values of measured parameters of inulin hydrogels. Tukey’s test at a significant level of α = 0.05 revealed significant differences between inulin gels without and with various concentrations of SA and CH.

## 3. Results and Discussion

It was discovered that the incorporation of SA as well as CH, regardless of concentration, had no effect on the gel forming ability of inulin. Under the studied circumstances (20 g/100 g INU, 0.1, 0.3, 0.5 g/100 g of SA and CH, respectively), the obtained water suspension developed a homogenous hydrogel structure (VGI = 100%, Table 1).

**Figure 1:**
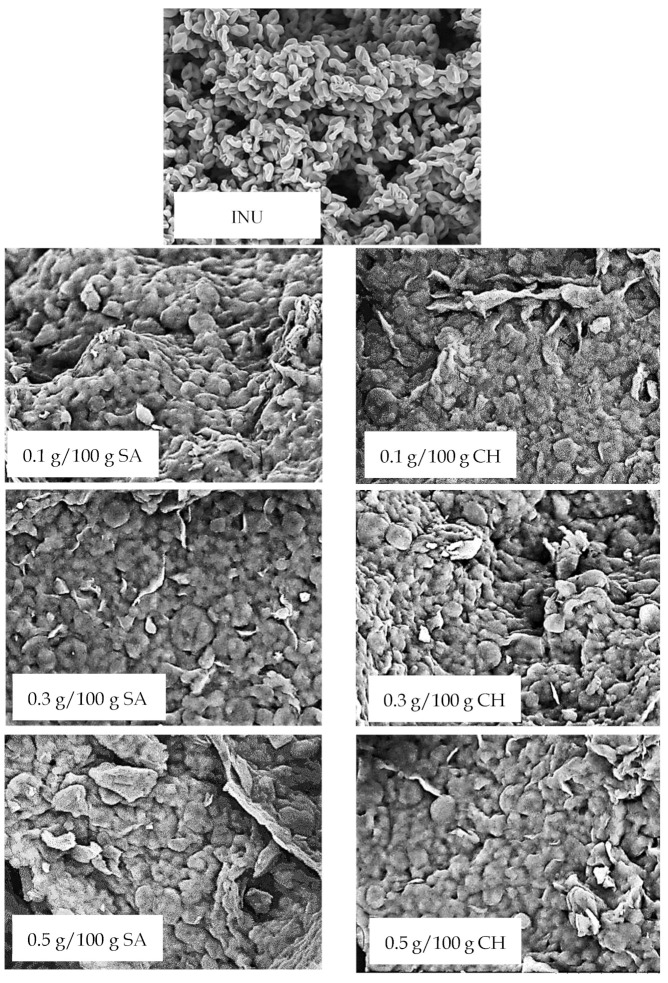
Effect of sodium alginate (SA) and chitosan (CH) addition on the microstructure of inulin hydrogels (magnification ×3000).

In order to investigate the structure of the obtained gels and visually review the effect of SA and CH on the microstructure of inulin hydrogels, electron microscopy was utilized (Figure 1). The control sample’s (INU) microstructure was characterized as a sponge-like structure due to the presence of inulin aggregates. However, the samples containing SA or CH (at all concentrations) revealed a more uniform microstructure, which appeared to be smoother and more coherent with slightly more closed pores when compared to the control sample. The inclusion of 0.3 g/100 g CH resulted in the most homogeneous gel structure with the smallest number of perforations. The presence of the chitosan caused an increase in the continuity of the gel structure. The same observation was made by Coa et al. [13], who investigated chitosan and inulin blend films. The authors noticed that by adding the inulin the microstructure was rougher, which was caused by the formation of bigger aggregates. On the contrary, chitosan formed a smoother microstructure. Moreover, alginate–guar gels also showed a similar compact and rigid microstructure [35]. In researches based on starch, authors have discovered that the addition of sodium alginate caused SA adhesions on the surface of starch granules and aggregations to form large particles, which increased with SA concentration [22]. This might be due to the ability of SA to penetrate the starch network, and of the cross-linking of both polysaccharides through hydrogen bonding. Generally, the addition of SA resulted in a denser networks, which led to a higher mechanical strength of gels [36].

In order to characterize the properties of the obtained gels, yield stress measurement was carried out. The yield stress value is the maximum shear stress that could be applied to a gel before it starts to flow [37]. In all cases, the addition of SA and CH to inulin hydrogels considerably impacted the yield stress of the investigated hydrogels, resulting in a substantial increase in the value of the tested parameter (Table 1). The increase in the yield stress value was directly proportional to the increasing concentration of the tested polysaccharides. With the addition of 0.3 and 0.5 g/100 g, the gel structure was strengthened to a greater extent by the addition of CH relative to SA, which resulted in significantly higher yield stress parameters. For the hydrogels with the addition of 0.5 g/100 g SA, the yield stress value reached 493.6 Pa, whereas when 0.5 g/100 g CH was added the yield stress value reached 745.3 Pa. The increased yield stress value might be due to the synergistic intermolecular interactions between the polysaccharides [38]. According to the literature, gel structures are considerably changed with SA concentration. According to Li et al. [39], for SA concentrations exceeding 0.3–0.5 g/100 g, the microstructure form becomes ordered and regular network became disordered. Other authors also observed a significant strengthening of the structure of gels as a result of an increase in CH concentration in the system. According to Yang et al. [40], an increase in CH concentration allows a higher degree of intermolecular interactions to occur and more energy to be stored in the system.

Analyzing the effect of SA and CH additions on the texture of inulin hydrogels, their strength and spreadability were investigated. The strength parameter represents the amount of force required to penetrate the structure of the studied hydrogel [41]. It was found that the addition (0.1, 0.3, and 0.5 g/100 g) of SA as well as CH increased the strength of inulin hydrogels (Table 1). This influence might be due to the formation of a more compact structure visible in the microstructural SEM images (Figure 1). The highest observed strength value was, in the case of SA addition, for 0.3 g/100 g (0.64 N), whereas the addition of 0.5 g/100 g SA to the inulin hydrogel caused a decrease in the strength. The decrease of strength of gels with SA addition was also reported by Yang et al. [38], who analyzed starch gels. They reported that SA addition to a rice starch matrix caused a weaker structure formation due to both polysaccharides competing for water. In the case of CH, a proportional increase in strength was observed along with an increase its concentration in the system (from 0.37 N for 0.1 g/100 g CH to 1.29 N for 0.5 g/100 g CH). Moreover, it was found that with the addition of CH at the level of 0.3 and 0.5 g/100 g, the induced hydrogels were characterized by greater strength than those with the addition of SA at respective concentrations. Similarly, Gonçalves et al. [42] observed that the increase of the concentration of CH is directly associated with the hydrogel strength [42], whereas Li et al. [39] reported that increasing the SA concentration in gels might cause the weakening of the structure. The structure of the gels was also analyzed in terms of their spreadability (Table 1). It was found that the addition of SA as well as CH at the lowest analyzed concentration of 0.1 g/100g had no significant effect on the spreadability of inulin hydrogels. Introducing more of these polysaccharides into the system, i.e., 0.3 g/100 g, resulted in a significant increase in spreadability (from 2.89 N * s for INU to 4.32 N * s for SA and to 3.71 N * s for CH). This indicated that the resulting hydrogels were less spreadable. However, the addition of the analyzed polysaccharides in their highest concentrations (0.5 g/100 g) had a different effect on the spreadability of gels, i.e., SA in the amount of 0.5 reduced the spreadability (to 3.44 N * s), while CH caused a further increase to this parameter (to 6.16 N * s). The higher spreadability values in the case of CH addition in comparison to SA can be caused by the formation of a stronger gel structure influenced by the increase of the attraction forces between the molecules. The effect of such a synergistic gelation on the spreadability of hydrogels was also reported in the case of protein and inulin combination [11,43], and in the case of other polysaccharides’ combinations, such as okra polysaccharides and kappa-carrageenan [44].

**Figure 2:**
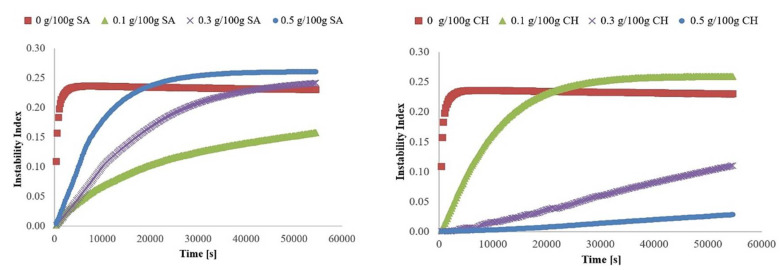
Influence of sodium alginate (SA) and chitosan (CH) addition on the destabilization of inulin hydrogels as a function of time.

An important parameter characterizing the gels is their stability. In order to determine the effect of the addition of sodium alginate and chitosan on this property of inulin hydrogels, the instability index was determined. It is a parameter that ranges from 0 for a stable gel structure to 1 for unstable gel. It was found that among the tested polysaccharides, the addition of chitosan had a greater impact on the stability of the formed gels than sodium alginate (Table 1 and Figure 2). Its addition, addition of amounts of 0.3 and 0.5 g/100 g resulted in a significant increase in the stability of gels, both in relation to gels containing only inulin (lowering the instability index from 0.23 (INU) to 0.11 for 0.3 g/100 g CH and to 0.03 for 0.5 g/100 g CH) and for inulin–alginate gels. In the case of gels containing sodium alginate, no significant differences in their stability with regards to INU gels were found. Only a tendency to slightly increase the stability of gels with the addition of 0.1 SA was observed, which is illustrated by the charts of destabilization kinetics (Figure 2). Despite the lack of studies regarding the stability of SA and CH inulin hydrogels, similar studies suggest that SA can retard the retrogradation and gelatinization of wheat starch, causing the formation of weak gels [45], which might explain the lack of SA impact on the stability of inulin hydrogels. On the other hand, the highest stability and compact microstructure of the inulin hydrogel with the addition of 0.3 and 0.5 g/100 g CH (Figure 1) might indicate the existence of an attraction between these biopolymers.

**Figure 3:**
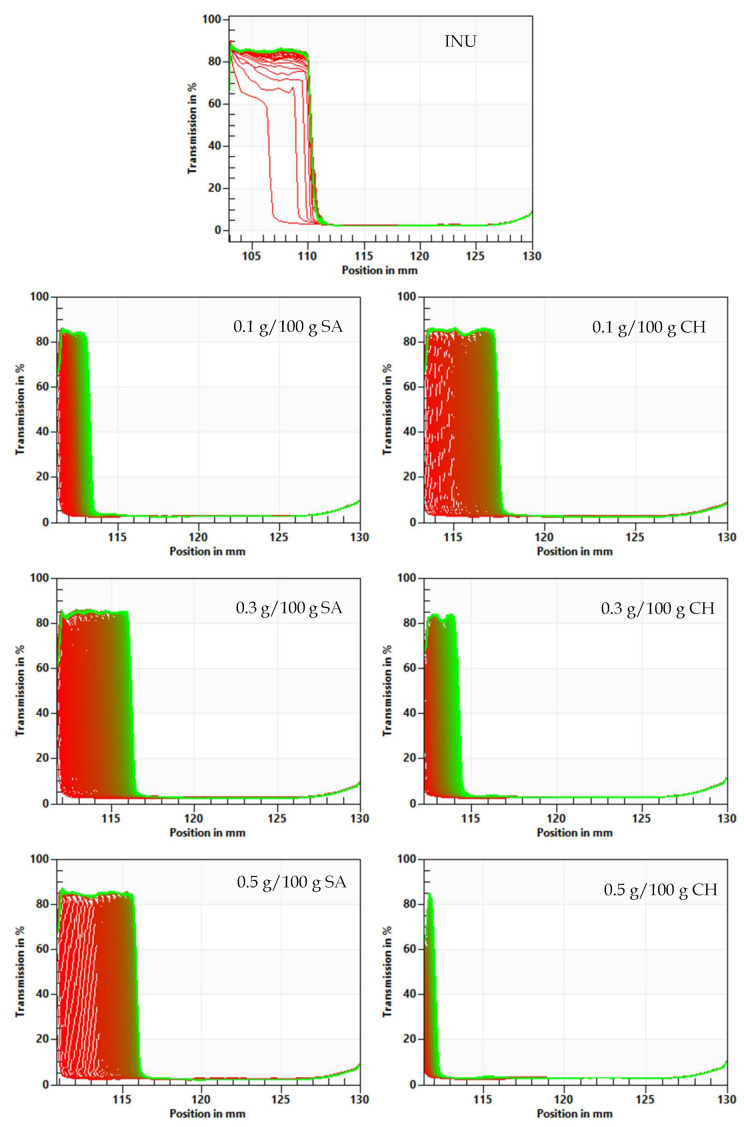
Influence of sodium alginate (SA) and chitosan (CH) addition on the transmission profile “fingerprints” of inulin hydrogels.

The “fingerprint” transmission profiles reflect the changes in a particle’s concentration inside the analyzed sample based on the STEP technology (space-time resolved extinction profiles). The evolution of extinction profiles’ “fingerprints” contains detailed information on the kinetics of concentration changes caused by phase separation. Low transmission indicates a high particle concentration, whereas high transmission indicates a low particle concentration. By examining the transmission profile (Figure 3), the destabilization behavior of hydrogels was investigated. The transmission profiles of all of the tested hydrogels had a similar shape (transmission at 80%), indicating that particles were moving towards the bottom of the sample because of the particle compression process. Based on the transmission profiles, it can be concluded that the compression process occurred the fastest in the control sample due to the weak inulin hydrogel structure. This finding is in accordance with other research on the stability of inulin hydrogels [11,32]. In the samples with the addition of 0.1 g/100 g CH, 0.3, and 0.5 g/100 g SA the sedimentation process was at a slower rate than in the case of the INU hydrogel (control sample). The slowest particles movement was observed in inulin hydrogel with the addition of 0.5 g/100 g CH, whereas the addition of 0.3 g/100 g CH and 0.1 g/100 g SA showed a similar effect on the sedimentation rate.

Analyzing the effect of the addition of the compared polysaccharides on the color of inulin gels, it was found that the introduction of 0.1–0.3 g/100 g sodium alginate to the inulin sol resulted in obtaining gels that were significantly brighter compared to gels containing only inulin (increase in the L * color parameter value from 88.54 (INU) to 89.56 for 0.1 g/100 g SA and 89.89 for 0.3 g/100 g SA) (Table 2). The opposite effect was found when 0.1 chitosan was introduced into the inulin sol. The obtained gels had significantly lower brightness (L * 87.97) than the INU and SA gels. This finding is in accordance with the study of Khanal et al. [46], who reported that the addition of SA to a low-fat cheddar cheese caused an increase of the L* parameter. Similar results were also found by Kowalczyk and Baraniak [47], who discovered that the addition of chitosan in lower concentration (0.25 g/100 g) caused a darkening of the edible pea protein films, while higher chitosan concentration (0.5 g/100 g) lowered the brightness of tested films.

In the case of the value of the color parameter a *, it was observed that the inclusion of the analyzed polysaccharides resulted in significantly lower values of the a * parameter in the produced hydrogels (Table 2). When CH was added to the inulin hydrogels, the a * color parameter dropped by 50% when compared to inulin hydrogels containing SA. According to Khoshgozaran-Abras et al. [48], the a * color parameter for a pure chitosan film is −3.82 ± 0.08. The results of this research support the conclusion that adding chitosan to inulin gels causes a decrease in the a * parameter. A similar decrease in the a * value was also reported in the study of sodium alginate (SA) addition as a fat substitute in cheddar cheese [46]. However, no significant change in the value of a * was reported when SA was used as a coating material for strawberries [49].

Analyzing the effect of the addition of sodium alginate and chitosan on the b * color component, it was found that the addition of the tested polysaccharides to the inulin sol resulted in a significant reduction in the value of this color parameter in relation to the gel containing only inulin (INU) (Table 2). Moreover, it was found that the addition of chitosan had less influence on the value of this color parameter (lowering the b * color parameter from an average of 2.23 (INU) to 1.06 (0.1 g/100 g CH) relative to that of sodium alginate (lowering the value to 0.50 (0.1 g/100 g SA)). However, opposite results were reported in the available literature stating that the addition of SA to low-fat cheddar cheese increased the value of b * [46]. According to Khoshgozaran-Abras et al. [48], the b * parameter of chitosan films decreased with the CH concentration in functional films, which is in accordance with the observed effect that it had on the b * parameter of inulin hydrogels.

**Figure 4:**
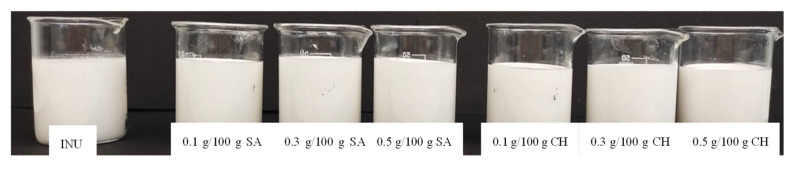
Sample photos showing the color of inulin gels obtained without and with various SA and CH concentrations.

To conduct a more detailed analysis on the influence of SA and CH addition on the color of hydrogels, the total color difference parameter (ΔE) was computed. It was found that in most cases the difference in the color of inulin hydrogels made without and with the addition of the compared polysaccharides was small and could only be seen by experienced observers (1 < ΔE < 2) (Table 2 and Figure 4). Only for the inulin gel containing 0.1 g/100 g of sodium alginate, the total color difference parameter in comparison with the INU was greater than 2.0, which means that also inexperienced observers can notice the difference in the color of the gels.

## 4. Conclusions

The addition of sodium alginate and chitosan to inulin solutions, regardless of concentration, had no effect on the ability of inulin to form a gel structure. Nonetheless, these additions had a significant impact on the properties of the obtained hydrogels, resulting in a more compact, smooth, and cohesive microstructure. In consequence, through the addition of these polysaccharides, the obtained gels were characterized by higher yield stress, strength, and spreadability than gels containing only inulin. At the same time, the addition of chitosan had a greater effect than sodium alginate in strengthening the structure of hydrogels, especially with the highest concentration (0.5 g/100 g). Chitosan also increased the stability of the gels. The obtained results indicate that through the addition of sodium alginate and chitosan, the properties of inulin hydrogels can be modified, and thus the inulin gels will be better adapted to the characteristics of the pro-health food products in which they will be used.

## Figures and Tables

**Table 1:** Effect of sodium alginate (SA) and chitosan (CH) addition on the physical properties of inulin hydrogels.

	Addition [g/100 g]	VGI [%]	Yield Stress [Pa]	Strength [N]	Spreadability [N * s]	Instability Index
INU	-	100 ^a^	195.0 ^a^ ± 20.0	0.24 ^a^ ± 0.01	2.89 ^a^ ± 0.01	0.23 ^cd^ ± 0.02
SA	0.1	100 ^a^	256.7 ^b^ ± 3.2	0.31 ^b^ ± 0.01	3.17 ^ab^ ± 0.25	0.16 ^bc^ ± 0.09
	0.3	100 ^a^	302.2 ^c^ ± 3.1	0.64 ^d^ ± 0.01	4.32 ^d^ ± 0.04	0.24 ^cd^ ± 0.01
	0.5	100 ^a^	493.6 ^d^ ± 11.8	0.42 ^c^ ± 0.00	3.44 ^b^ ± 0.07	0.26 ^d^ ± 0.00
CH	0.1	100 ^a^	278.0 ^bc^ ± 5.0	0.37 ^b^ ± 0.01	2.75 ^a^ ± 0.12	0.26 ^d^ ± 0.02
	0.3	100 ^a^	438.0 ^d^ ± 11.0	0.83 ^e^ ± 0.00	3.71 ^c^ ± 0.07	0.11 ^ab^ ± 0.01
	0.5	100 ^a^	745.3 ^e^ ± 7.0	1.29 ^f^ ± 0.03	6.16 ^e^ ± 0.09	0.03 ^a^ ± 0.01

All values are mean with standard deviation (n = 3). According to Turkey’s test, ^a–f^ values followed by the same letter within the column do not differ significantly (*p* > 0.05).

**Table 2:** Effects of sodium alginate (SA) and chitosan (CH) addition on the color parameters of inulin hydrogels and the total color difference (ΔE).

	Addition [g/100 g]	L *	a *	b *	ΔE ^#^
INU	-	88.54 ^b^ ± 0.02	−0.44 ^d^ ± 0.03	2.23 ^e^ ± 0.05	-
SA	0.1	89.56 ^c^ ± 0.47	−0.85 ^c^ ± 0.02	0.50 ^a^ ± 0.02	2.05
	0.3	89.89 ^c^ ± 0.21	−0.87 ^c^ ± 0.03	1.17 ^c^ ± 0.06	1.77
	0.5	88.59 ^b^ ± 0.11	−0.78 ^c^ ± 0.03	0.85 ^b^ ± 0.02	1.42
CH	0.1	87.97 ^a^ ± 0.28	−1.84 ^a^ ± 0.03	1.06 ^c^ ± 0.03	1.91
	0.3	88.30 ^ab^ ± 0.19	−1.51 ^b^ ± 0.05	2.08 ^d^ ± 0.08	1.11
	0.5	88.06 ^ab^ ± 0.16	−1.53 ^b^ ± 0.07	2.03 ^d^ ± 0.03	1.21

Values are presented as mean with standard deviation (n = 3). According to Turkey’s test, ^a–e^ values followed by the same letter within the column do not differ significantly (*p* > 0.05). ^#^ Total color difference parameter calculated in relation to INU.
